# Mapping Half a Century of Global Brain Abscess Research: A Bibliometric Analysis of Output, Themes, and Collaboration (1975–2025)

**DOI:** 10.7759/cureus.114519

**Published:** 2026-08-14

**Authors:** Varun Gopal Rajan, Trayi Joshi

**Affiliations:** 1 Neurosurgery, Adichunchanagiri Institute of Medical Sciences, Bengaluru, IND; 2 Medical Education, Neuroglia Health Private Limited, Bengaluru, IND; 3 School of Medicine, Adichunchanagiri Institute of Medical Sciences, Bengaluru, IND

**Keywords:** bibliometric analysis, brain abscess, central nervous system infection, cerebral abscess, neurosurgery, openalex

## Abstract

Brain abscess is an uncommon but life-threatening intracranial infection managed across neurosurgery, infectious diseases, microbiology and neuroradiology, for which no single unified international guideline exists; despite a large literature, the research field has been mapped only once, in a single-database study of 994 title-matched records. We performed a comprehensive bibliometric analysis of global brain-abscess research, retrieving journal articles (1975-2025) from the OpenAlex database using an exact-phrase title/abstract search and computing performance metrics, bibliometric laws and science mapping in R. Across 9,097 articles (35,138 authors; mean 4.5 authors per article; 12.6% single-authored), annual output grew at 4.07% per year (95% CI 3.78-4.36; quasi-Poisson regression), a doubling time of 17.4 years, with the 2020-2025 window already approaching the entire 2010s; this rate is statistically indistinguishable from the growth of the scientific literature as a whole, and the field’s share of world output fell between 1975 and 2015 before recovering after 2020. Output was led by the USA, India, Japan, China and the UK, with modest international co-authorship (7.2%), and publications spanned neurosurgical, infectious-disease, paediatric and microbiological journals. Themes shifted from computed tomography toward magnetic resonance imaging, retained a persistent microbiology-antibiotics-meningitis core, and showed the recent emergence of cardiology-related themes consistent with the endocarditis and cyanotic-congenital-heart-disease associations; author productivity was highly dispersed (88.8% single-paper authors), open access rose to 75% of recent output, only 6% of articles recorded funding, and Indian institutions led productivity. All metrics were regenerated from an archived copy of the dataset rather than the live database, which is continuously updated; relevance was validated by independent dual screening of a random sample (Cohen’s kappa = 0.95), and findings were robust in a title-only sensitivity analysis and cross-validated across three bibliographic databases. Brain-abscess research is sustained, distributed across venue types and internationally, with substantial output from institutions in lower- and middle-income settings. These findings describe publication patterns rather than research quality or clinical impact, derive principally from a single database, and identify questions for future work rather than recommendations for practice.

## Introduction and background

Brain abscess is a focal suppurative infection of the brain parenchyma. It remains uncommon, but it is dangerous, and the people who manage it come from very different clinical worlds - neurosurgeons drain it, infectious-disease physicians and microbiologists treat the organism, neuroradiologists characterise it, and paediatricians and cardiologists meet it as a complication of other disease. Management has changed substantially over five decades: cross-sectional imaging, image-guided stereotactic aspiration, and evolving antimicrobial strategy have all reshaped practice [1,2]. Yet a recent survey of specialists still describes wide variation in care and the lack of a single unified guideline [3].

A large body of literature has accumulated, but its shape has not been described [4]. Which countries and groups drive it? Where is it published? How have its themes moved? Bibliometric analysis answers these questions by treating the published record as data [5]. One previous bibliometric study of brain-abscess research has been reported [6]. It retrieved 994 records published from 1980 onward carrying the phrase in the title, restricted to the Science Citation Index and Science Citation Index-Expanded within Web of Science, and described distributions of publication year, journal, language, publisher, country, first author, institution, subject category, funding and citations. The present study differs in retrieval and in analytic scope: the phrase is matched in title or abstract rather than title alone and retrieval is not restricted to a citation-index subset, giving 9,097 records against 994 and extending the window back to 1975; and the analysis adds co-word and thematic-evolution mapping, collaboration-network analysis, the bibliometric productivity laws, and open-access and funding profiles, together with a formally validated relevance screen. We set out to map five decades of it: output over time, the most productive journals, authors, countries and institutions, the thematic structure and its evolution, and the international collaboration network.

## Review

Methods

**Figure 1:**
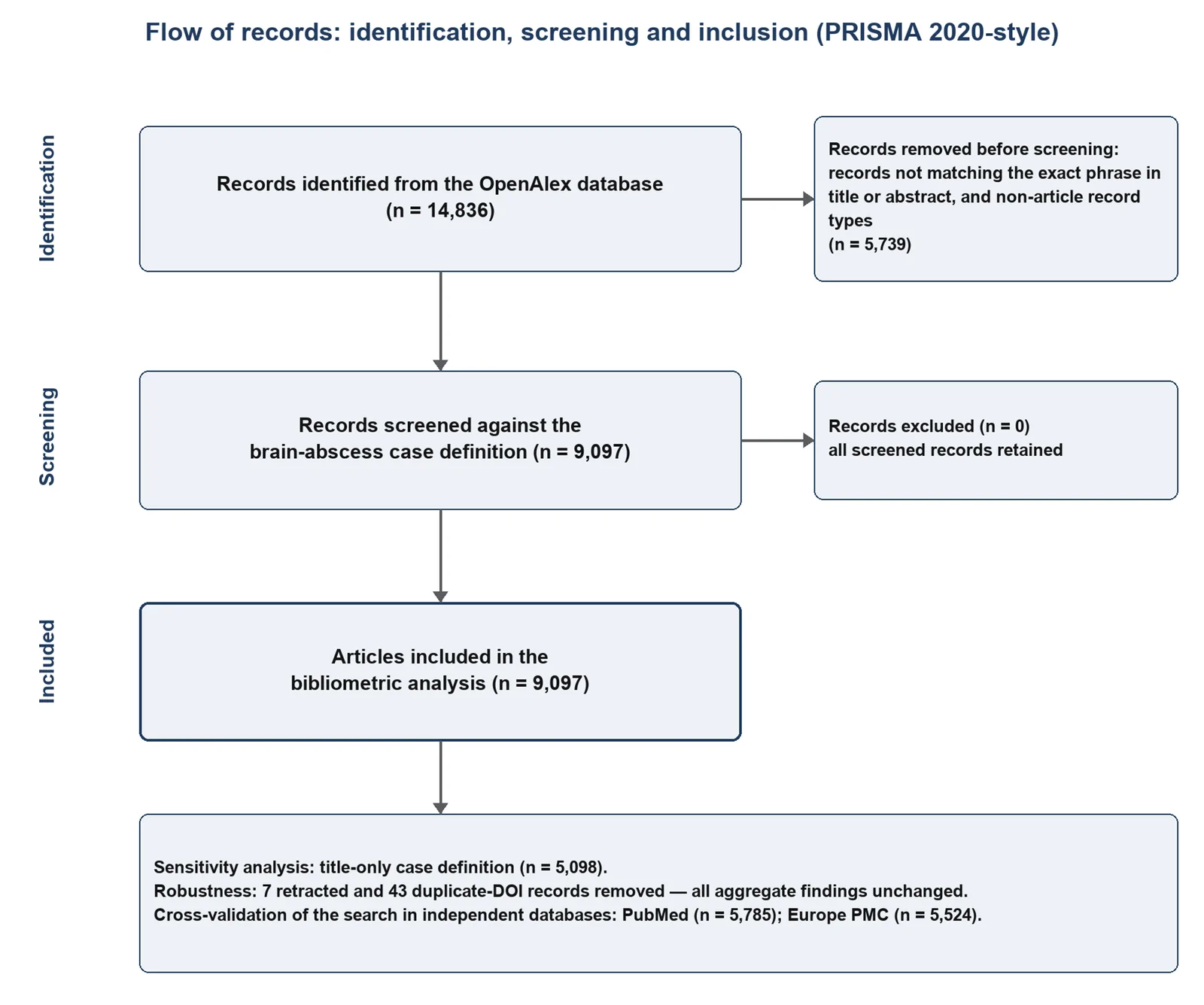
Flow of records through identification, screening and inclusion (PRISMA 2020-style).

We searched OpenAlex, an open, comprehensive bibliographic database [7], retrieved with the openalexR package [8]; OpenAlex offers reference coverage comparable to the proprietary Web of Science and Scopus databases for recent literature [9]. The query used exact-phrase matching in the title or abstract ("brain abscess", "cerebral abscess" or "intracranial abscess"), restricted to journal articles published 1975-2025 and run on 29 June 2026; an initial word-level query was deliberately tightened to exact phrases. Records containing an exact phrase in the title or abstract formed the primary set (n = 9,097); a title-only set (n = 5,098) was retained as a sensitivity analysis (Figure 1). Year, source, citation and concept fields were taken from the standardised record fields, and author names and institution countries were parsed directly from the structured authorship data of each record (each author's primary-institution country was used; per-document countries were de-duplicated). Performance analysis and science mapping were conducted in R using the bibliometrix package [10], following established bibliometric methodology [5], including bibliometric laws (Bradford, Lotka, Price), collaboration indices, productive institutions, open-access and funding profiles, and a keyword-based thematic map (Callon centrality-density). All reported metrics were regenerated from the raw data (a full reproducibility audit reconciled every figure); topic relevance was assessed by independent dual screening of a random 100-record sample against a pre-specified written rubric, with inter-rater agreement quantified and discordances resolved by consensus; the search was cross-validated against equivalent title/abstract queries in PubMed and Europe PMC; and retracted and duplicate-DOI records were identified and tested for influence (Figure 1). The study used only published bibliographic metadata, involved no human participants, and required no ethical approval.

Relevance screening was performed independently by both authors, each blinded to the other's assignments, against a written classification rubric (version 1.1) frozen before either reviewer opened the sample. Agreement was quantified using Cohen's kappa for the three-category classification and two pre-specified collapsed contrasts, with 95% confidence intervals and a prevalence- and bias-adjusted kappa; discordant records were resolved by discussion to consensus, and the basis for each decision was recorded. Three further sensitivity analyses, excluding records flagged during screening as title-only, tie-break or long compendium abstracts, had been specified in advance but could not be performed because the free-text flag field was not preserved when the second reviewer's assignments were collated; this is reported as a deviation from the pre-specified plan.

Publication trend was modelled by quasi-Poisson regression of annual article counts on year with a log link, the quasi-Poisson family being chosen because annual counts were overdispersed (dispersion 3.22) and a Poisson model would yield falsely narrow intervals. Acceleration was tested by adding a quadratic term in year and comparing models by F-test, refitting across restricted windows (1975-2019, 1990-2025, 2000-2025) as a sensitivity analysis. To assess the field's share of world output, the model was refitted with the logarithm of total annual OpenAlex article counts, retrieved under the same document-type filter, as an offset. Proportions are reported with Wilson score 95% confidence intervals. A novelty search was run on 31 July 2026 in PubMed and OpenAlex using ("brain abscess" OR "cerebral abscess" OR "intracranial abscess") AND (bibliometric* OR scientometric* OR "research trends" OR "publication trends" OR "knowledge mapping" OR "science mapping" OR "citation analysis"), with no date or language restriction, verifying in each database that both halves of the query returned results independently. All data, screening logs and analysis scripts are openly available at the Open Science Framework [11].

The query was run through the openalexR interface to the OpenAlex API as oa_fetch(entity = "works", title_and_abstract․search = '"brain abscess" OR "cerebral abscess" OR "intracranial abscess"', from_publication_date = "1975-01-01", to_publication_date = "2025-12-31", type = "article"). Quotation marks enforce exact-phrase matching and OR combines the three synonyms; the title-only sensitivity set was retrieved with the identical call substituting title․search for title_and_abstract․search. Non-article record types, including book chapters, datasets, dissertations, preprints and peer-review records as classified by the database, were excluded at the query level by the type = "article" parameter rather than filtered afterwards.

No language filter was applied, because excluding non-English literature would bias a study whose aims include describing global and lower- and middle-income contribution. The cost of that choice is disclosed here: matching is applied to the indexed title and abstract, so a record published in another language without an English title or abstract cannot match an English-phrase query and is under-captured. The database assigns one record per work, so no exact record-level duplicates were present; residual duplication was tested at DOI level, identifying 43 records (0.5%) sharing a DOI and 7 (0.08%) flagged as retracted. These were retained in the primary analysis, and every headline metric was re-run with all 50 removed, leaving the growth rate, mean citations, open-access share and top-five country ranking unchanged. Where metadata were missing, analyses used pairwise deletion with no imputation, and the relevant denominator is reported alongside each affected statistic.

This database was chosen as the primary source for four reasons: it is openly licensed, so the search can be repeated by any reader without subscription access; its reference coverage has been shown comparable to the major subscription databases for recent literature [9]; it indexes a broader range of venues, including the open-access and non-MEDLINE journals in which a substantial share of contemporary brain-abscess case literature appears; and it exposes structured authorship, institution, funding and open-access fields in a single record, which the collaboration and funding analyses require. Databases were not merged, because cross-database record linkage carries its own error profile and would prevent readers reproducing the dataset with a single query; a single primary source was instead cross-validated against two independent databases.

Bibliometric indices follow their standard definitions: the Bradford zone-1 core is the smallest set of sources that together account for one-third of all articles, author productivity is compared against Lotka's inverse-square law, and the Price threshold is taken as the square root of the author count. The concept co-occurrence network was clustered with the Louvain algorithm using a fixed random seed (set․seed(7)) and laid out with the Fruchterman-Reingold algorithm (set․seed(42)); the thematic map follows the Callon centrality-density formulation implemented in bibliometrix; and the 100-record validation sample was drawn with set․seed(2026). Fixing these seeds makes the clustering, layout and sampling exactly reproducible.

Global output and growth

**Figure 2:**
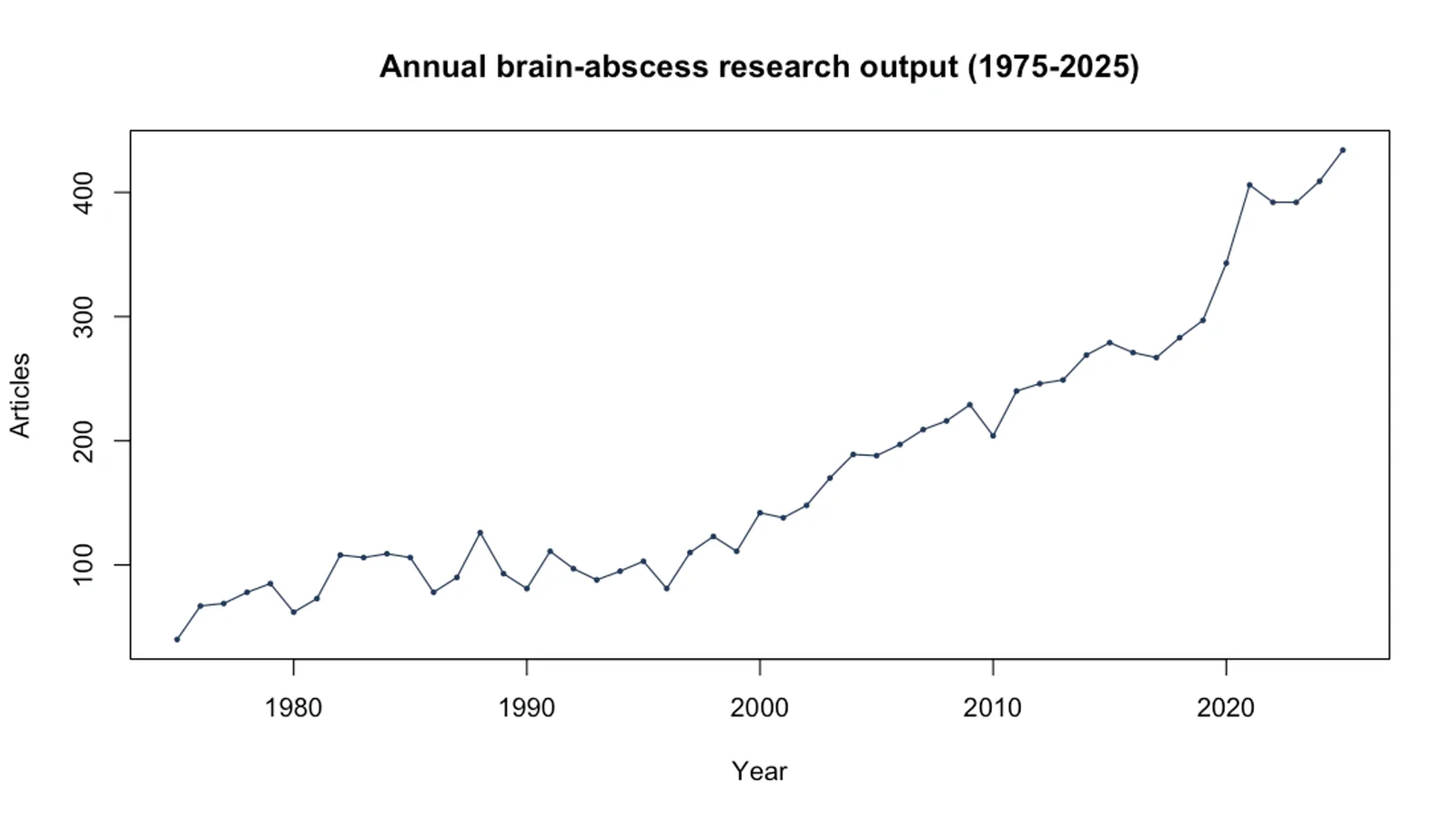
Annual brain-abscess research output, 1975–2025.

The 9,097 articles spanned 1975-2025 across 2,312 journals, with 35,138 distinct authors, a mean of 4.5 authors per article and 12.6% single-authored papers. Output rose steadily - 339 articles in the 1970s, then 951, 1,000, 1,826 and 2,605 in the following decades - with the 2020-2025 window already holding 2,376 articles, close to the whole of the 2010s (Figure 2). Quasi-Poisson regression of annual counts on year yielded a growth rate of 4.07% per year (95% CI: 3.78-4.36), corresponding to a doubling time of 17.4 years (95% CI: 16.2-18.7). A quadratic term testing for a change in growth rate was significant over the full window (F = 10.35, p = 0.002) but did not persist in any window beginning in 1990 or later, reversing sign in 1990-2025; with residual autocorrelation also present (lag-1 r = 0.44), the trend is best described as sustained approximately exponential growth rather than acceleration.

Growth relative to the scientific literature

The observed growth rate is statistically indistinguishable from the 4.10% annual growth and 17.3-year doubling time reported for modern science as a whole [12]. Modelled against total annual OpenAlex article output as an offset, brain-abscess publications declined as a share of world output between 1975 and 2015 (-1.89% per year, 95% CI -2.26 to -1.53, p < 0.001), falling from a peak of 10.2 articles per 100,000 world articles in 1982 to a trough of 3.8 in 2010, before recovering to 6.2 by 2025. Growth in absolute publication counts therefore tracks the expansion of the scientific literature rather than a disproportionate increase in attention to intracranial abscess.

Productive journals

**Figure 3:**
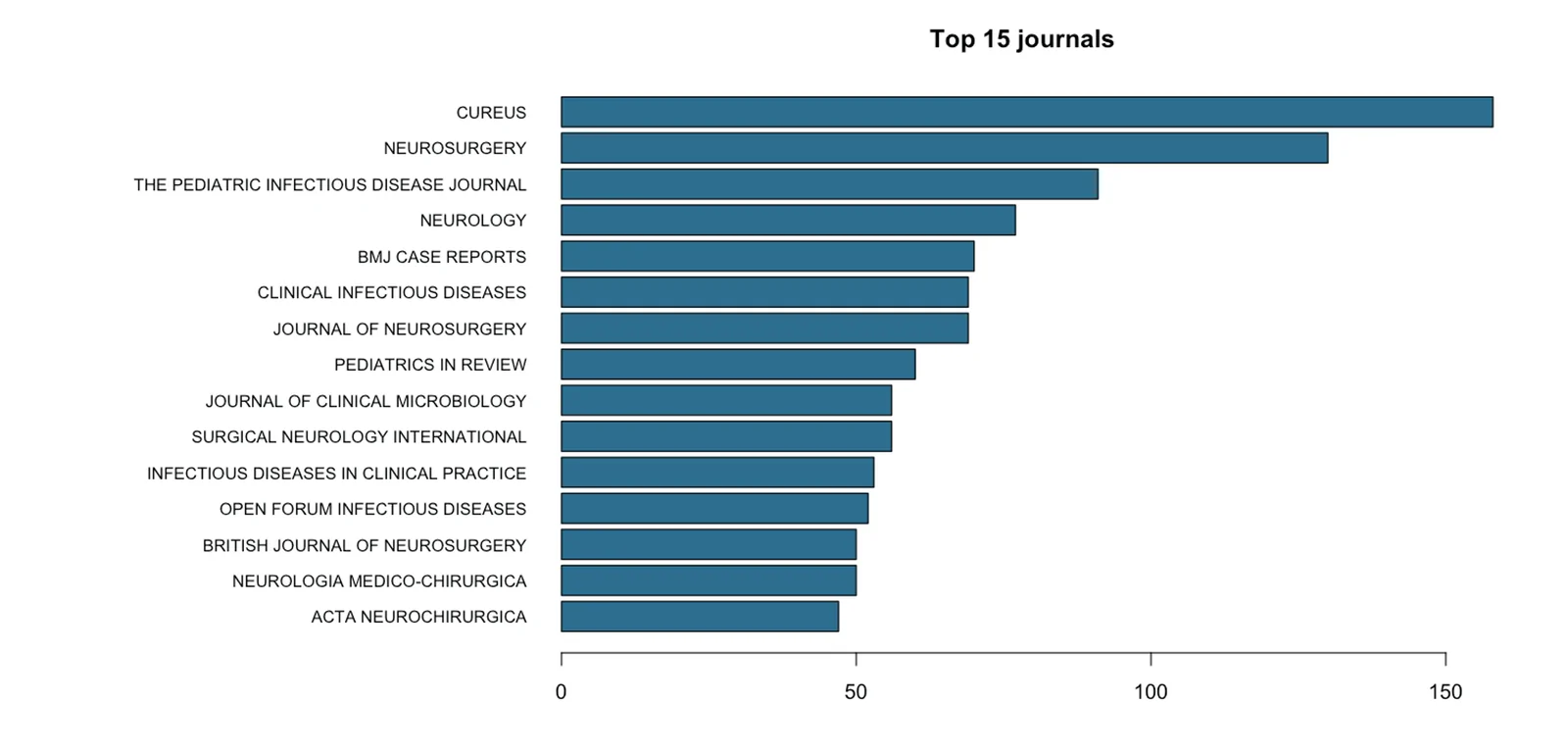
Most productive journals.

**Figure 4:**
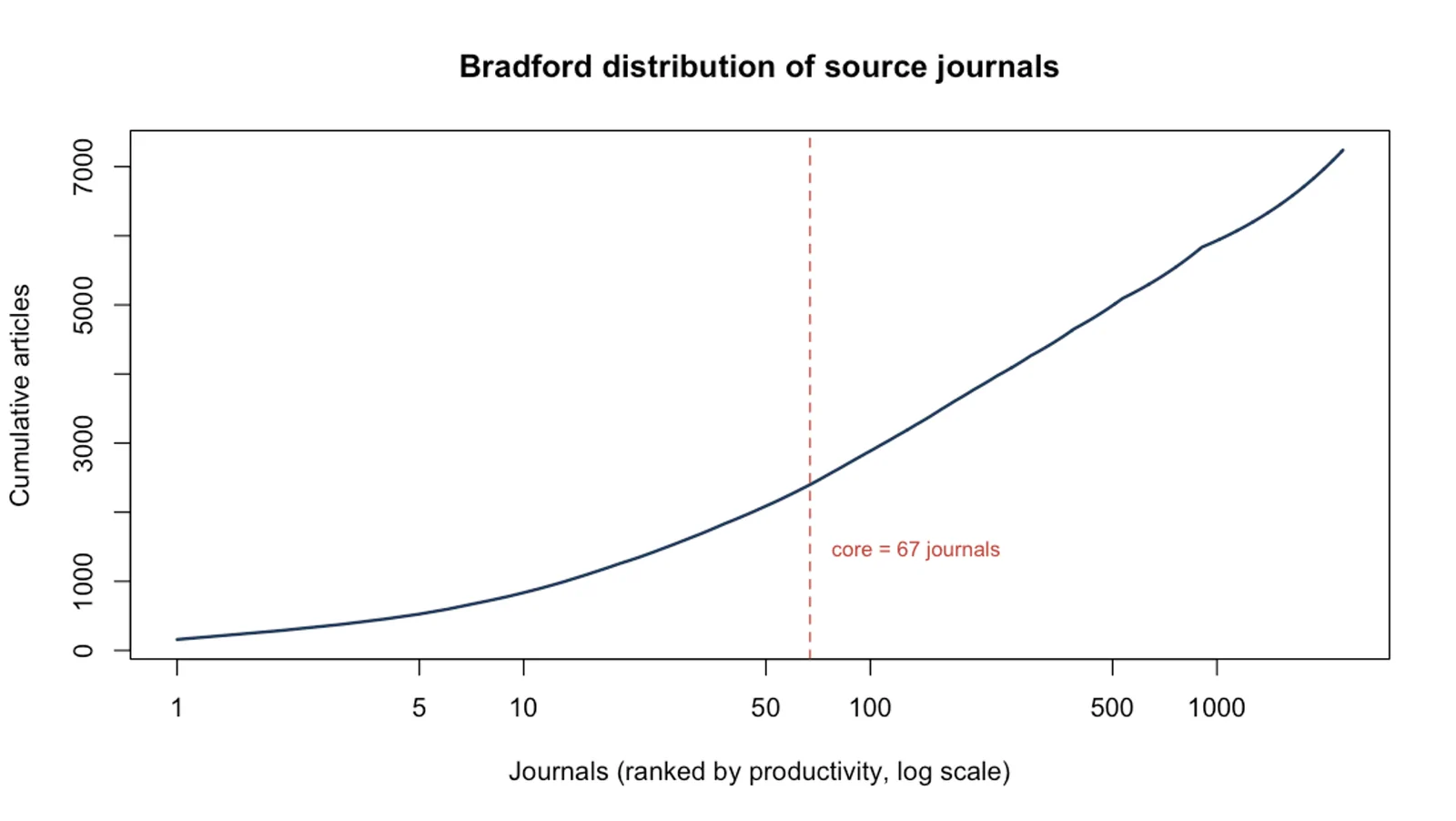
Bradford distribution of source journals (core ≈ 67 journals).

Publication was distributed rather than concentrated, reflecting the condition's cross-disciplinary nature. The most productive venues were Cureus (158), Neurosurgery (130), The Pediatric Infectious Disease Journal (91), Neurology (77), BMJ Case Reports (70), Clinical Infectious Diseases (69), the Journal of Neurosurgery (69), Pediatrics in Review (60), the Journal of Clinical Microbiology (56) and Surgical Neurology International (56) (Figure 3). The sources followed a Bradford distribution: a core of just 67 of the 2,309 journals carried roughly one-third of all articles (Figure 4).

Contributing countries and institutions

**Figure 5:**
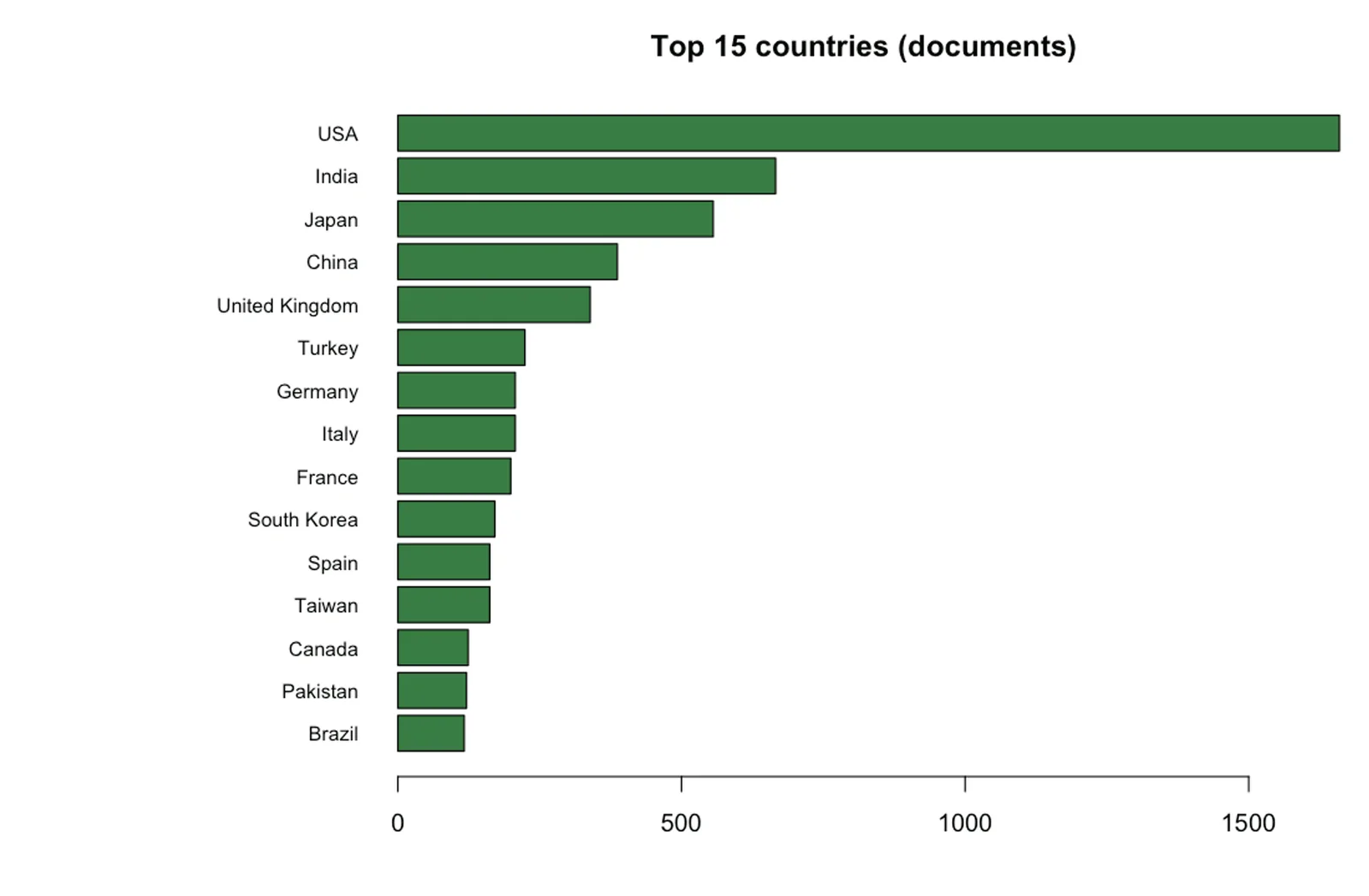
Most productive countries (by documents).

**Figure 6:**
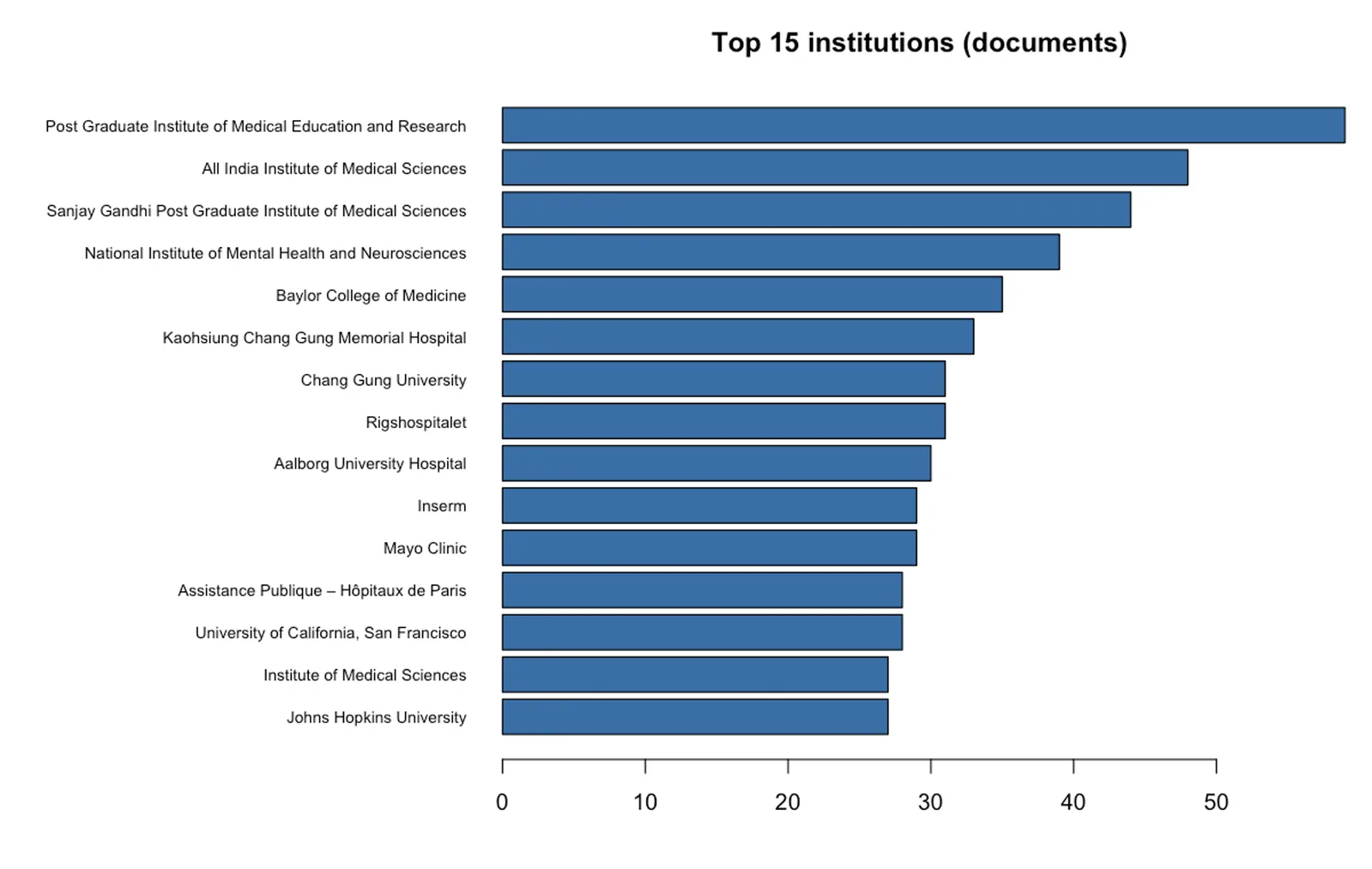
Most productive institutions

Country metadata were available for 73.7% of articles. Output was led by the USA (1,660), followed by India (666), Japan (556), China (387), the UK (339), Turkey (224), Germany and Italy (207 each), France (199) and South Korea (171); India's second-place position and the visible contribution of lower- and middle-income settings are notable features of this field (Figure 5). The most productive institutions were led by Indian centres - the Postgraduate Institute of Medical Education and Research (59), the All India Institute of Medical Sciences (48), Sanjay Gandhi Postgraduate Institute of Medical Sciences (44) and the National Institute of Mental Health and Neurosciences (39) - followed by Baylor College of Medicine, the Chang Gung centres (Taiwan), Rigshospitalet and Aalborg University Hospital (Denmark), Mayo Clinic and Johns Hopkins University (Figure 6).

Authors, productivity and collaboration

**Figure 7:**
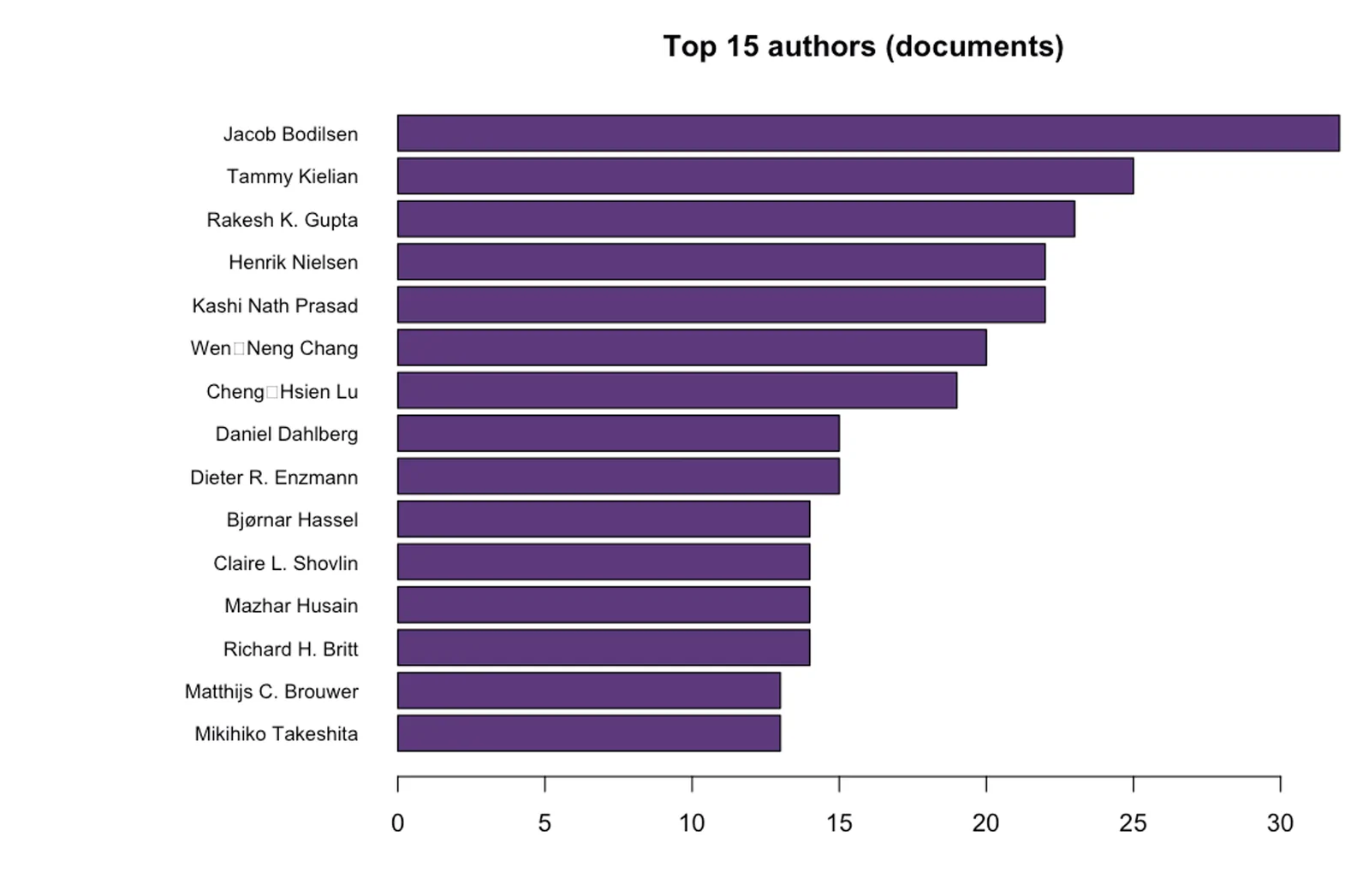
Most productive authors (by documents).

**Figure 8:**
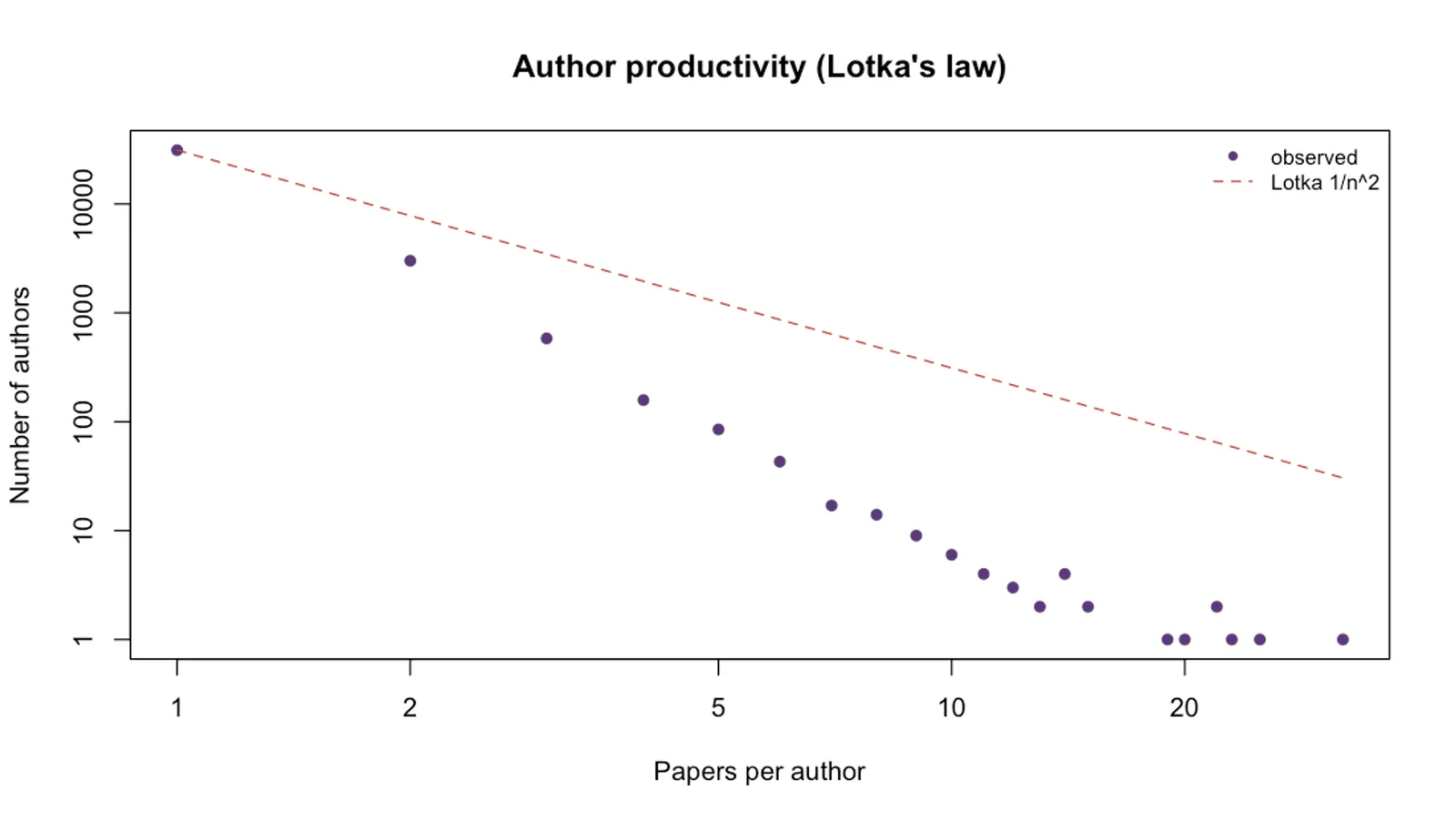
Author productivity distribution (Lotka’s law).

**Figure 9:**
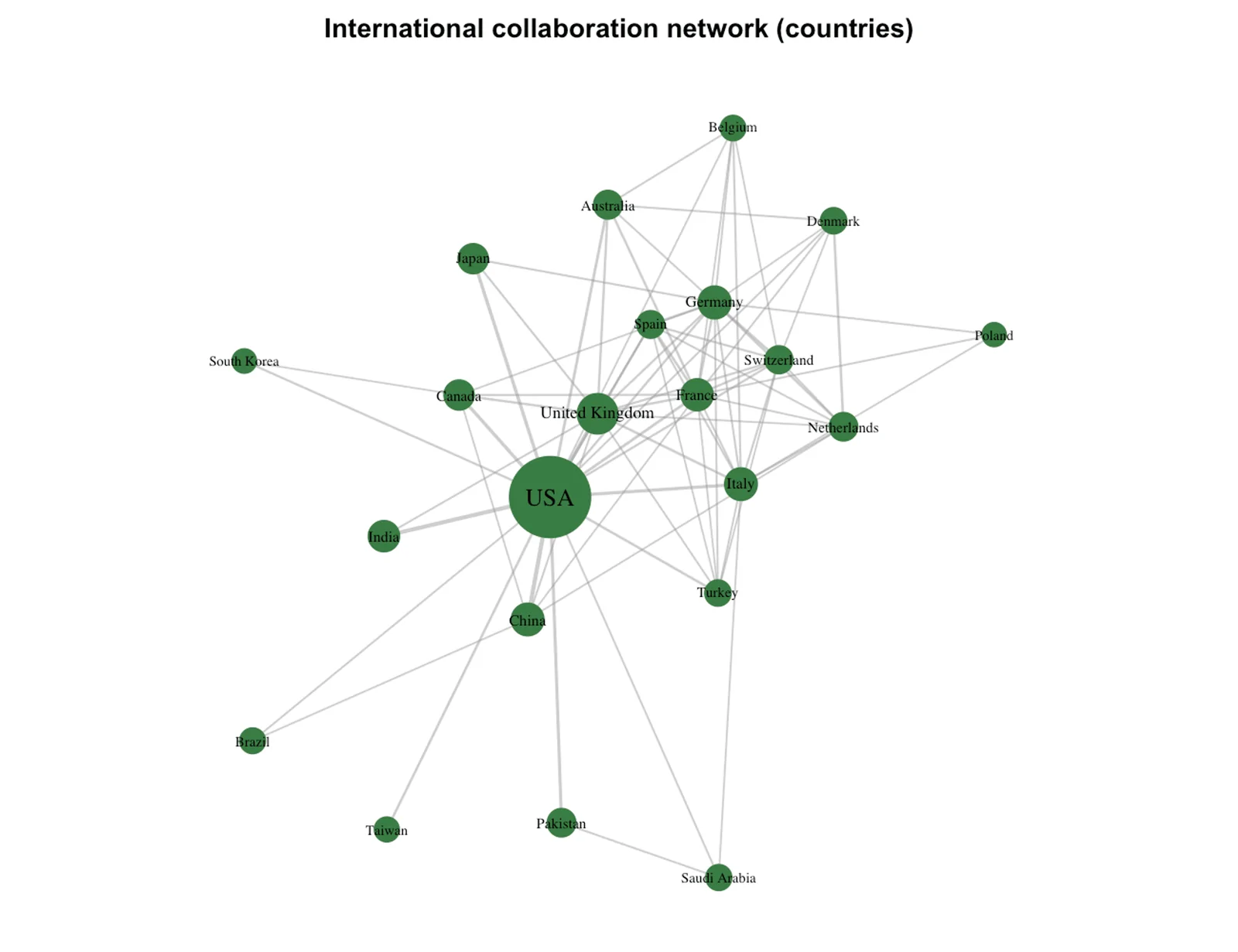
International collaboration network (countries).

The most productive authors were established figures in brain abscess research, including J. Bodilsen (32), T. Kielian (25), R. K. Gupta (23), H. Nielsen (22), and K. N. Prasad (22) (Figure 7). Productivity was highly dispersed: 88.8% of the 35,138 authors contributed a single article - well above the ~60% expected under Lotka's law (Figure 8) - and the most prolific cohort (the 187 authors corresponding to the square root of the author count) appeared on only 7.6% of papers, so Price's law of a concentrated elite did not hold. Collaboration was nonetheless the norm (degree of collaboration 0.86; collaboration index 5.07; mean 4.5 authors per article). International co-authorship was modest at 7.2%, with the USA as the central hub most strongly linked to the UK, Germany, China, Italy, France and India (Figure 9).

Thematic structure and evolution

**Figure 10:**
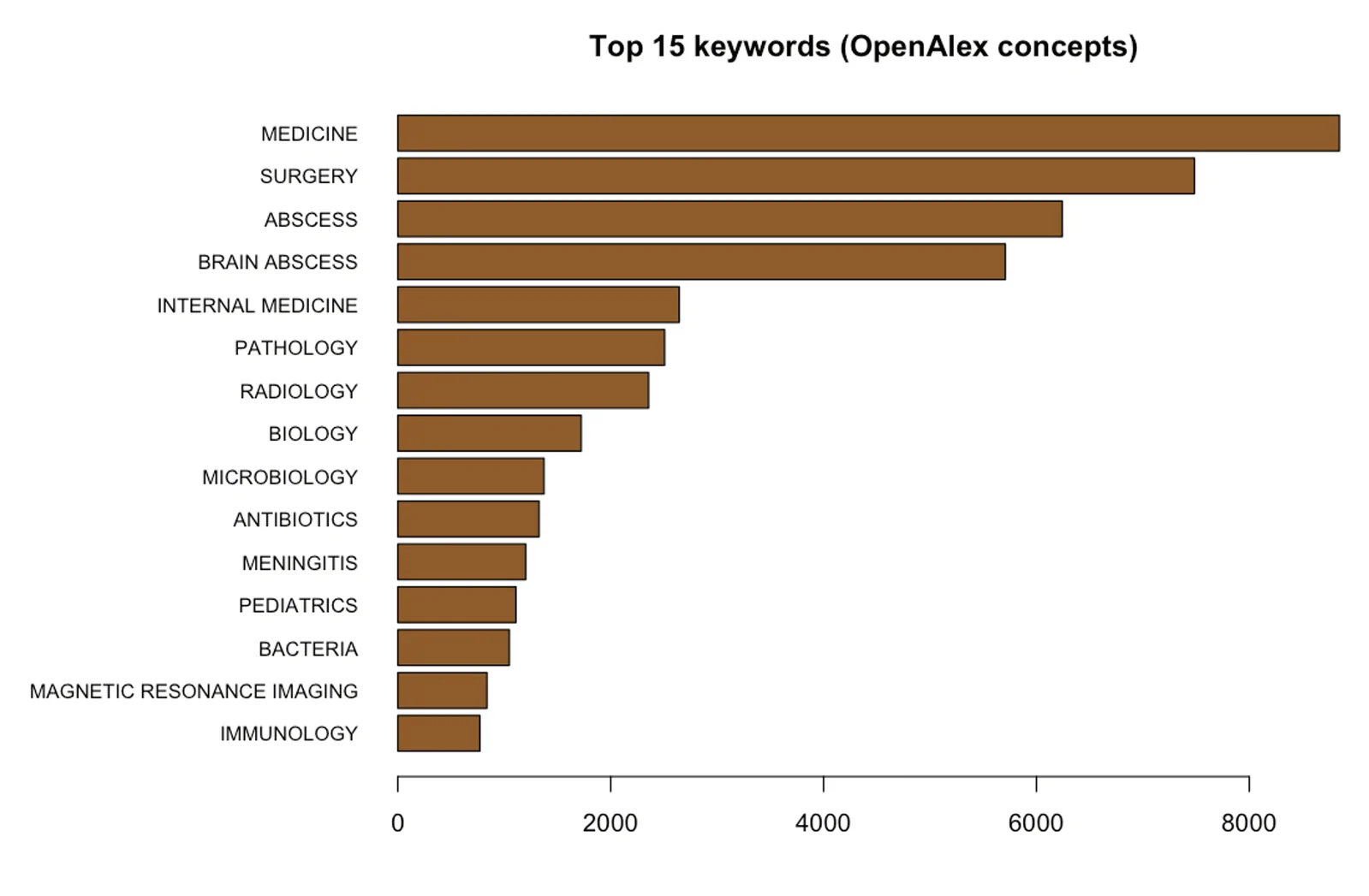
Most frequent concepts.

**Figure 11:**
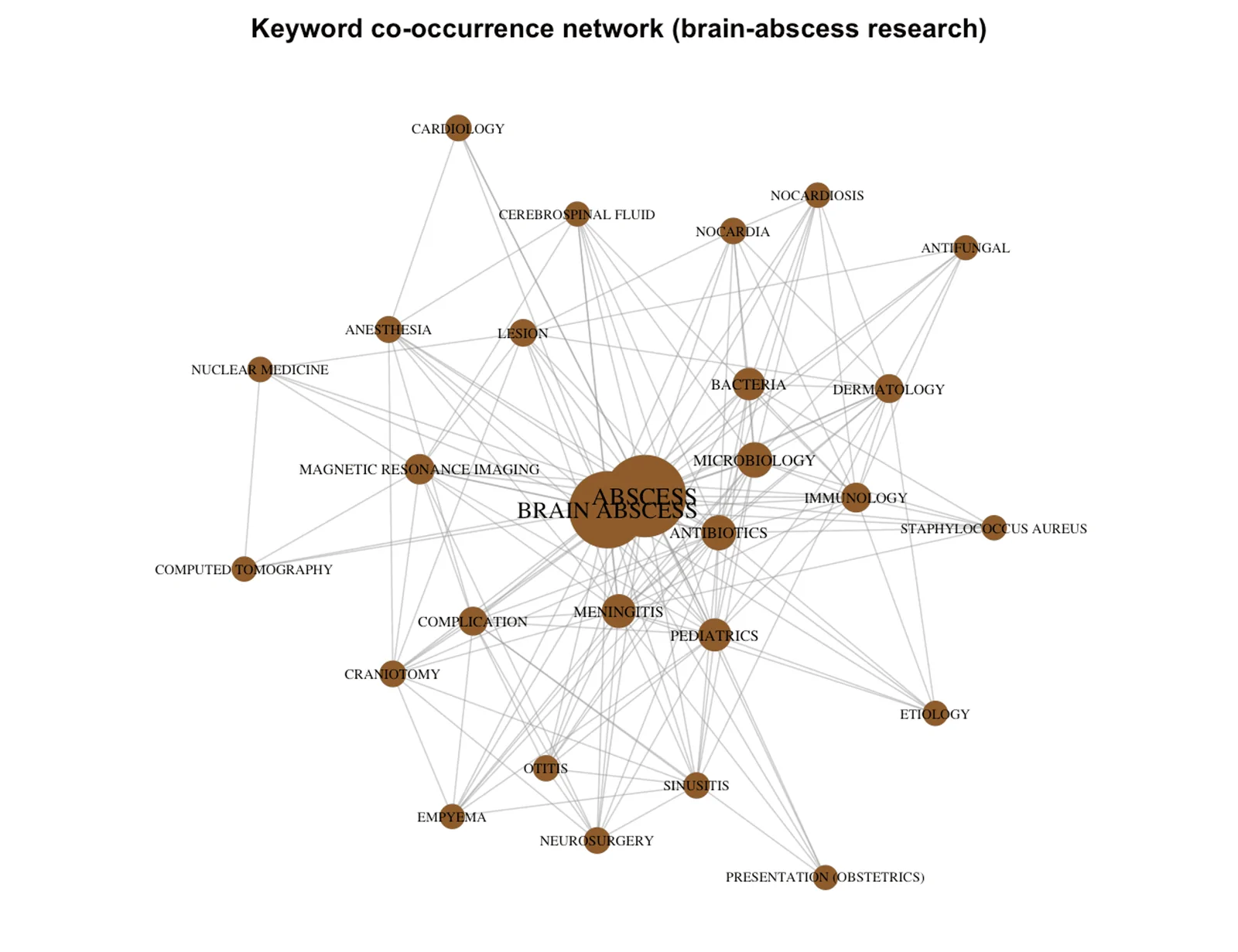
Concept co-occurrence network.

**Figure 12:**
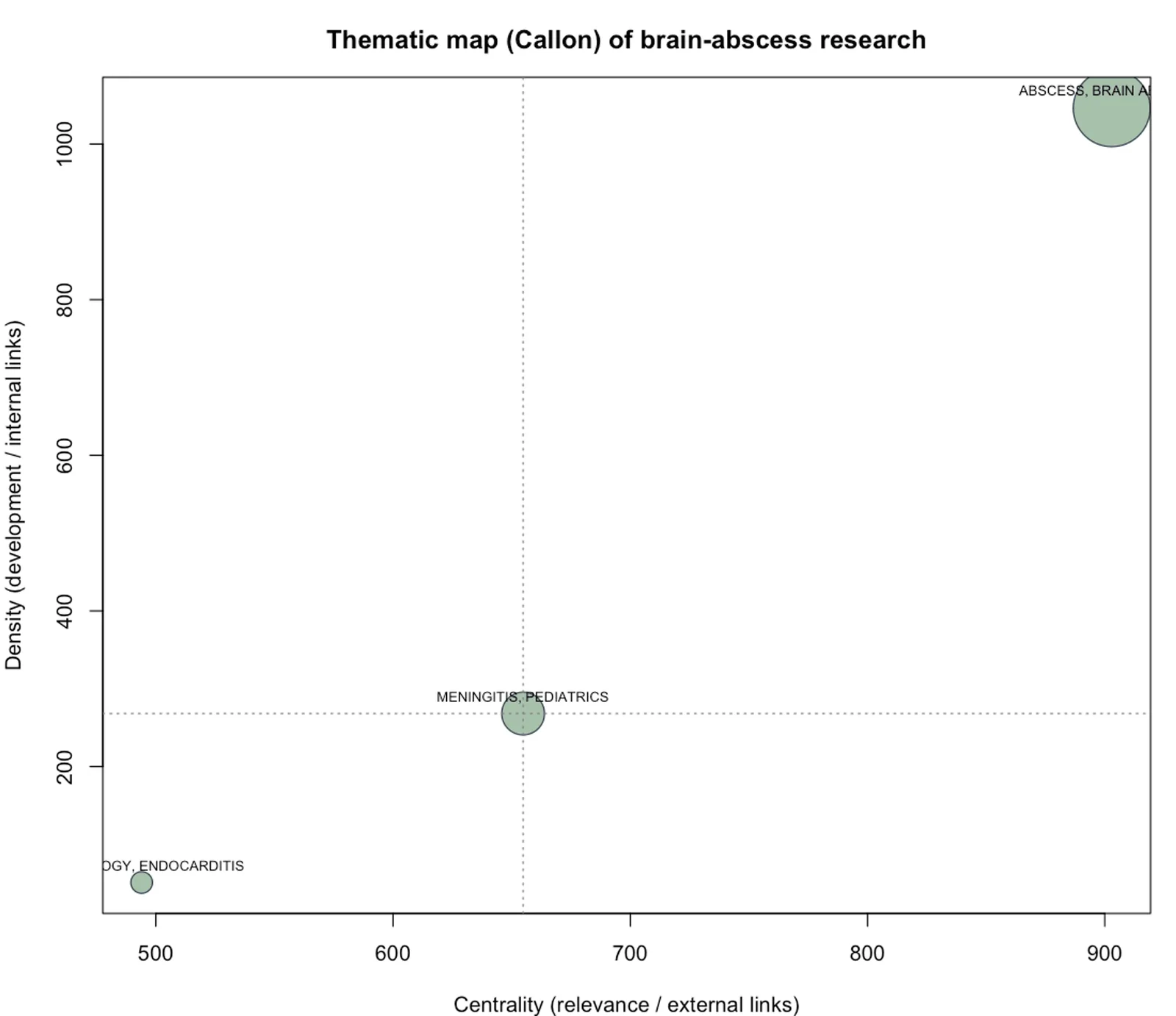
Thematic map (Callon centrality–density).

Beyond the core concepts (abscess, brain abscess, antibiotics, microbiology, meningitis, bacteria), the thematic structure shifted across three periods (Figures 10-11). Computed tomography dominated the imaging vocabulary of 1975-1999, while magnetic resonance imaging rose to prominence from 2000 onward, mirroring the introduction of diffusion-weighted imaging to distinguish abscess from cystic tumour [13]. Paediatrics and immunology remained consistently present, and in the most recent period (2013-2025), cardiology-related themes emerged, consistent with the recognised associations between brain abscess, infective endocarditis and cyanotic congenital heart disease. A thematic map (Callon centrality-density; Figure 12) resolved this structure into three clusters: a central, well-developed motor theme of abscess management and antibiotic therapy; a transversal meningitis-paediatrics-complications theme; and a distinct but less-developed emerging cluster of cardiology, endocarditis and heart disease, quantitatively locating the cardiac association as the field's growing edge.

Most-cited work

The most-cited documents were predominantly broad central nervous system infection and imaging papers - including reviews of CNS infection, neurological complications of infective endocarditis [14], and diffusion-weighted imaging discrimination of abscess from tumour [13] - reflecting the field's anchoring in imaging and systemic disease associations.

Open access and funding

**Figure 13:**
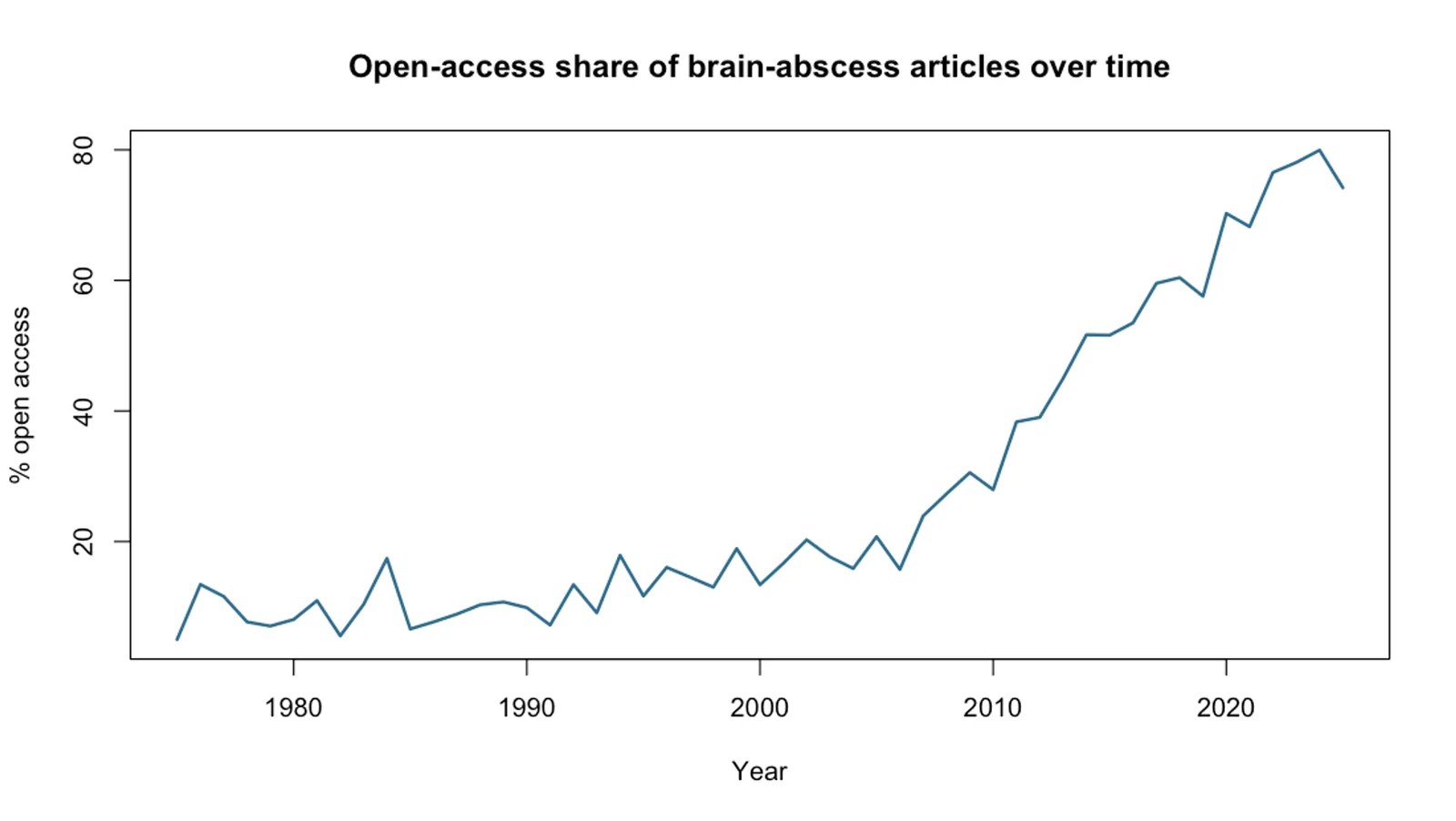
Open-access share of articles over time.

Open access rose steadily, from under 10% before 2000 to 75.4% in 2020-2025 (40.6% overall; Figure 13). Funding metadata were sparse: only 6.0% of articles recorded any funder, most frequently the US National Institutes of Health, the National Natural Science Foundation of China and the Indian Council of Medical Research, consistent with a field advanced largely through unfunded clinical scholarship.

Cross-validation, precision and robustness

Equivalent title/abstract queries in two independent biomedical databases returned concordant counts - PubMed 5,785 and Europe PMC 5,524 - both close to the OpenAlex title-only set (5,098) and below the broader primary set (9,097), indicating that the larger OpenAlex count reflects its broader venue coverage rather than spurious inclusion. A random 100-record sample was screened independently by both authors against a written rubric frozen before either reviewer opened the sample, with each record assigned to one of three categories: intracranial abscess as the primary subject; substantive intracranial-abscess content within a record whose main subject was another condition; or no substantive intracranial-abscess content. Agreement across the three categories was 97.0% (Cohen's kappa = 0.95, 95% CI 0.89-1.00); for the collapsed contrast of primary subject against all other records, kappa was 0.98 (95% CI 0.94-1.00). The three discordant records were resolved by discussion to consensus. On the consensus classification, 58.0% of records (95% CI 48.2-67.2) had intracranial abscess as their primary subject and 71.0% (95% CI 61.5-79.0) were primarily on-topic or contained substantive intracranial-abscess content. Binomial intervals are Wilson intervals. Findings were also robust to a stricter title-only case definition (n = 5,098), which reproduced the growth rate, authorship structure, citation density, the identical 7.2% international co-authorship rate and the country ranking; and to the removal of 7 retracted (0.08%) and 43 duplicate-DOI (0.5%) records, which left every aggregate finding unchanged.

Discussion

Brain-abscess research is growing steadily, in step with science as a whole, and is unmistakably multidisciplinary. That the field has merely kept pace with global scientific growth, and lost ground as a share of it for three decades, is itself a finding. Its output is not concentrated in a handful of specialist journals but spread across neurosurgery, infectious diseases, paediatrics and microbiology - a structural reflection of how the disease is actually managed, by teams rather than a single speciality.

The geography is striking: the USA leads, but India ranks second and lower- and middle-income settings are well represented, a pattern that fits the higher burden of predisposing conditions (untreated otogenic and sinogenic infection, cyanotic congenital heart disease) in these regions [1], and argues for research and guidelines relevant beyond high-income centres. The thematic trajectory tracks technology and pathophysiology - the move from CT to MRI vocabulary, and the recent surfacing of cardiology themes alongside the long-standing endocarditis and cyanotic-heart-disease associations [14]. These shifts describe indexed terminology, are compatible with documented clinical associations, and may also reflect changes in concept assignment within the database over time. Collaboration, however, is low: a 7.2% international co-authorship rate, in a globally distributed field with no unified guideline, is an opportunity rather than a verdict.

Two structural features reinforce this. Author productivity is unusually dispersed - nearly nine in ten authors contributed a single article, and the most prolific cohort accounts for under 8% of output - and recorded funding is almost absent (6%). A globally distributed, largely unfunded, weakly collaborative field with no unified guideline is precisely the setting in which an international collaborative registry could change practice. The leadership of Indian and other lower- and middle-income institutions, together with the surge in open access (now three-quarters of recent output), is an asset for exactly such an equitable, coordinated effort. The emergence of a distinct cardiac thematic cluster describes an active publication area and a candidate topic for focused clinical review; a keyword cluster cannot support a clinical recommendation.

Limitations

This analysis used a single primary database (OpenAlex); coverage and indexing differ between databases, and roughly a quarter of records lacked structured country metadata. OpenAlex institution disambiguation is imperfect, so a small number of authors and institutions may be mislabelled or merged. Concept tags are relatively coarse, so the thematic map describes broad structure rather than fine-grained topics. Finally, bibliometrics describe research activity and visibility, not clinical quality or correctness.

Four further limitations attach to the validation and trend analyses. The earlier relevance assessment judged relevance primarily by titles and did not retain per-record classifications, so it is not directly comparable to the dual screen reported here; the present figures are reported on their own terms and not as a replication or correction of that assessment. One of the two screeners performed that earlier assessment and could not be blinded to its aggregate result. Three pre-specified sensitivity analyses could not be performed because the screening flag field was not preserved during collation. Finally, total OpenAlex output was not monotonic after 2015, reflecting indexing and document-type classification rather than real change, so the share-of-output estimate is anchored to the stable 1975-2015 window, and the precision estimates describe only the retrieved set, carrying no information about records the search failed to retrieve.

## Conclusions

Across 50 years, brain-abscess research has grown into a multidisciplinary and globally distributed field, expanding at the same rate as the scientific literature at large, with notable contributions from India and other lower- and middle-income settings, a clear imaging-technology trajectory in the indexed terminology, and the recent emergence of cardiac themes. These findings describe publication patterns rather than research quality, clinical practice or disease burden, and derive principally from a single database. Whether a coordinated multi-country study can address the dispersed authorship, near-absent funding, and low international collaboration rate, and whether the evidence base can support the development of a unified guideline, are questions for future work using other study designs rather than the conclusions of this analysis.
